# Healthy eating and active living policy, systems, and environmental changes in rural Louisiana: a contextual inquiry to inform implementation strategies

**DOI:** 10.1186/s12966-023-01527-w

**Published:** 2023-11-13

**Authors:** Bailey Houghtaling, Laura Balis, Nila Pradhananga, Melissa Cater, Denise Holston

**Affiliations:** 1 School of Nutrition and Food Sciences, Louisiana State University (LSU) & LSU Agricultural Center, Baton Rouge, LA 70803 USA; 2 Department of Human Nutrition, Foods, and Exercise, Virginia Tech, Blacksburg, VA 24061 USA; 3 Gretchen Swanson Center for Nutrition, 14301 FNB Parkway, Suite 100, Omaha, NE 68154 USA; 4 Agricultural and Extension Education and Evaluation, Louisiana State University (LSU) & LSU Agricultural Center, Baton Rouge, LA 70803 USA

**Keywords:** Rural health, Cooperative Extension, Policy, systems, and environmental (PSE) change, Consolidated Framework for Implementation Research, Implementation science

## Abstract

**Background:**

Healthy eating and active living policy, systems, and environmental (PSE) changes are implemented across the United States through Cooperative Extension. However, translating multisector PSE changes to practice in community settings is challenging and there is a lack of knowledge about barriers and facilitators to PSE changes among state Extension systems using standardized frameworks. Therefore, a research-to-practice partnership effort aimed to identify Louisiana Cooperative Extension Service Family and Consumer Science (LFCS) practitioners’ barriers and facilitators to implementing PSE changes in rural Louisiana communities.

**Methods:**

A qualitative approach using the 2022 Consolidated Framework for Implementation Research (2022 CFIR) was used. Focus group discussions were conducted at five LFCS regional trainings between February and May 2022. All LFCS practitioners with any level of experience implementing healthy eating and active living PSE changes were eligible to participate, with emphasis on understanding efforts within more rural communities. Focus group discussions were audio-recorded and transcribed verbatim. Researchers analyzed qualitative data using the constant comparison method and 2022 CFIR domains and constructs including *Inner Setting* (LFCS organization), *Outer Setting* (rural Louisiana communities), *Innovation* (PSE changes), and *Individuals* (PSE change implementation actors/partners).

**Results:**

Across the five regions, LFCS practitioners (*n* = 40) described more barriers (*n* = 210) than facilitators (*n* = 100); findings were often coded with multiple 2022 CFIR domains. Reported *Inner Setting* barriers were lack of formal or informal information sharing and lack of access to knowledge and information. *Outer Setting* barriers included sustaining and initiating community partnerships and local environmental or political conditions. *Individual* barriers included a lack of time and expertise, and *Innovation* barriers included the complex nature of rural PSE changes. Facilitators were mentioned at multiple levels and included community partner buy-in and practitioners’ motivation to implement PSE changes.

**Conclusions:**

Implementation strategies are needed to build on organizational strengths and to overcome multi-level barriers to PSE change implementation among LFCS practitioners. The results from the in-depth contextual inquiry used could serve as a guide for future pragmatic assessment efforts among other state Extension systems or as a model for identifying barriers and facilitators and associated implementation strategies among other public health systems in the U.S. and abroad.

## Background

To mitigate persisting income and place-based health disparities described in Healthy People 2030 [1], healthy eating and active living policies, systems, and environmental (PSE) changes are needed in the United States (U.S.). The Dietary Guidelines for Americans 2020–2025 [2] recommends regular consumption of a variety of fruits, vegetables, and whole grains and the Physical Activity Guidelines for Americans [3] recommends 150 min of moderate to vigorous aerobic physical activity and two sessions of strength training each week. Traditional health promotion approaches have focused on individual health education and behavior change; however, without removing structural barriers many rural American households with lower socioeconomic status are less likely to meet healthy eating and active living guidance [4, 5].

Several federal organizations have authorized PSE changes for practice networks who are funded to deliver the U.S. Department of Agriculture’s Supplemental Nutrition Assistance Program Education (SNAP-Ed), Expanded Food and Nutrition Education Program (EFNEP), and the Centers for Disease Control and Prevention (CDC) Division of Nutrition, Physical Activity, and Obesity programs, for example [6–8]. These priorities for PSE changes aim to make healthy choices the default in local communities (e.g., healthy retail, complete streets policies, safe routes to school, workplace policies, and shared use agreements) and are increasingly implemented through the National Cooperative Extension System (Extension), a U.S. public health structure associated with land-grant universities in each state and territory that is over 100 years old [9–11].

Land-grant universities have a tripartite mission (teaching, research, Extension) and are charged with extending university resources to communities through the implementation of evidence-based interventions (EBI), which are estimated to reach over 6 million Americans each year [12]. While each state Extension system varies, this is typically accomplished through state-level specialists that are usually PhD-trained (housed in universities), county- or regional-level practitioners or paraprofessionals that are typically masters’ degree-trained (housed in county offices), and county educators that help to provide culturally relevant education and assist with PSE efforts [12]. These faculty and staff work within specific focus areas, with public health work frequently centered within Extension’s Family and Consumer Science (FCS) or Health and Nutrition initiatives [11, 13, 14]. For many years, FCS and similar Extension initiatives have successfully delivered individual/interpersonal level nutrition [15–18] and physical activity interventions [11, 19, 20]. More recently, Extension formalized PSE changes through their 2014 National Framework for Health and Wellness [10]; however, translating multisector PSE changes to practice in community settings is challenging [21–23].

For example, Extension county-based practitioners, who are responsible for selecting and implementing EBIs to meet identified community needs, have reported lower confidence to implement PSE changes compared to delivering traditional direct education [24, 25]. Lacking or nonexistent standardized training protocols to build capacity for PSE implementation is another prominent barrier reported by Extension practitioners [24, 25]. Challenges also exist in working with community partners and coalitions [26], including the multisector nature of this work and, in some cases, partners’ preferences for direct education interventions [27, 28]. Finally, among rural community settings in particular, there are often unique time and resource constraints (e.g., longer travel times) for Extension practitioners, partners, and community members in collaborating to implement PSE changes [24, 25, 27]. While there are examples of Extension practitioners overcoming barriers and successfully integrating PSE changes [14, 29–31], impacts have been limited and efforts have not yet penetrated state systems [14, 31].

For Extension to fully integrate PSE changes in state and national systems, implementation strategies or specific methods or techniques to improve EBI adoption, implementation, sustainment, and scaling [32–36] are needed. This aligns with Extension’s (2021) National Framework for Health Equity and Well-Being [37], regarding opportunities to use implementation science theories, models, and frameworks to understand contextual barriers and facilitators and the implementation strategies needed to overcome barriers and build on successes. Although barriers and facilitators have been previously reported, there is a lack of systematic knowledge about contextual factors that influence the implementation of healthy eating and active living PSE changes within state Extension systems [38]. For example, existing research has primarily focused on nutrition educators and paraeducators funded through specific sources (e.g., SNAP-Ed and EFNEP), rather than all eligible state delivery agents [25, 39–43], and often has not based on standard implementation science frameworks. This likely limits the transferability of results to other systems or contexts. As such, the use of a comprehensive implementation science framework focused on barriers and facilitators could support a more rapid contextual inquiry process to support PSE change implementation and scaling [44, 45].

Therefore, the purpose of this study was to identify Louisiana Cooperative Extension Service Family and Consumer Science (LFCS) practitioners’ barriers and facilitators to the implementation of healthy eating and active living PSE changes in rural Louisiana communities. This work represented a research-to-practice partnership within a statewide public health practice model to identify and tailor implementation strategy supports required for healthy eating and active living PSE change implementation and eventual scaling. The approach used in this study and the opportunities to apply contextual inquiry results to more rapid assessment methods, as we discuss, could serve as a model for similar work occurring in other public health systems in the U.S. and abroad.

## Methods

Healthy eating and active living PSE changes (herein: PSE changes) implemented by LFCS practitioners in rural communities occur at the intersection of individual, partnership, community, and social-political factors [24, 29, 46]. Therefore, a qualitative study using the updated 2022 Consolidated Framework for Implementation Research (2022 CFIR) [47] was used to elicit rich information about barriers and facilitators to implementing PSE changes at multiple levels. The 2022 CFIR is an ecologic and determinant implementation science framework that captures factors that influence implementation among multiple domains and associated constructs. The domains, and their operationalization for this study, are *Inner Setting* (LFCS organization), *Outer Setting* (rural communities), *Innovation* (PSE changes), and *Individuals *(implementation actors/partners) [47]. The 2022 CFIR *Implementation Process* domain was not a main focal point for this study. An exempt determination for research with human subjects was received from the Louisiana State University (LSU) Agricultural Center’s Institutional Review Board (IRBAG-21–0184).

### Setting

Louisiana Cooperative Extension Service, based at two land-grant university systems–LSU Agricultural Center and Southern University Agricultural Research and Extension Center (SUAREC)–is part of a national network of practitioners with a long history of bridging evidence-based practices and local community implementation [12]. LSU is a land-grant university established by the Morrill Act in 1862 [48] and SUAREC is a historically Black land-grant university established by the Second Morrill Act 1890 [49]. LFCS practitioners implement direct education (individual/interpersonal) and PSE changes throughout the state, with emphasis on improving food security and dietary quality among households with lower socioeconomic status given funding streams and associated priority outcomes (e.g., SNAP-Ed, EFNEP, and CDC).

Louisiana has a high proportion of households with children living in poverty and consistently ranks near last on population health metrics compared to other U.S. states [50]. Food and physical activity environment surveillance scores are also generally poor [50, 51]. LFCS practitioners also operate in many rural Louisiana communities [52], as rural residents with lower socioeconomic status have expressed built environment barriers to healthy practices [53]. At the time of this study, LFCS practitioners were implementing a variety of PSE changes among several rural Louisiana communities using a community coalition model [29, 38, 54]. There was an explicit goal among LFCS leaders to scale PSE change implementation to additional rural communities throughout the state, and eventually, statewide, which drove the focus of this research-to-practice project.

### Sampling and recruitment

A purposive sampling strategy was used to engage all LFCS practitioners operating in Louisiana parishes (equivalent to U.S. counties) at the time of this study. This included LFCS Regional Coordinators (regional leadership for Agents), Agents (professionals working at the local or parish level), and Educators (paraprofessional nutrition educators working at the local or parish level). While LFCS practitioners also work to implement PSE changes in urban Louisiana communities, this research explicitly focused on their efforts in more rural communities using local Extension guidelines for rural which follow standard (yet varying) classification schemes [52]. Two researchers (BH, NP) traveled to LFCS regional trainings occurring in each of the five Louisiana Extension regions (Central, Southwest, Southeast, Northeast, and Northwest) between February and May of 2022 to recruit all current LFCS practitioners to engage in focus group discussions. LFCS practitioners were not required to be currently implementing PSE changes at the time of the study to ensure broad perspectives related to barriers and facilitators.

### Data collection

Focus group discussions were chosen instead of individual interviews, as researchers assumed group interaction would facilitate the sharing and generation of ideas related to multi-level barriers and facilitators to LFCS practitioners’ implementation of PSE changes [47]. Semi-structured focus group guides were created (Table 1) following CFIR questionnaire guidance [55] and were reviewed for face validity by study partners within the Louisiana Extension system (MC, DH). LFCS practitioners operating in both urban and rural parishes/communities were encouraged to share experiences based on their work in the more rural locations, specifically. The goal for data collection was qualitative saturation (e.g., the “saturation” of ideas or concepts where no new information is shared), which has been shown to generally require between 4 to 8 focus group discussions [56].

**Table 1:** Example questions from a semi-structured focus group questionnaire following the Consolidated Framework for Implementation Research [55]

Please describe the healthy eating and active living policy, systems, and environmental (PSE) changes that have been implemented in rural Louisiana communities. a. What is the process you use for choosing, implementing, and sustaining the PSE strategies? Is it easy or difficult to get community buy-in for healthy eating and active living PSE changes? b. Which healthy eating and active living PSE strategies do you think would be a priority for your communities, if any?
Take a moment to consider challenges and opportunities regarding delivering healthy eating and active living PSE changes in rural communities (or what you might think as a barrier/facilitator if you were to start this type of programming in your communities). a. What aspects of healthy eating and active living PSE change interventions specifically are a barrier or facilitator to moving this type of work forward? b. What aspects of Louisiana Cooperative Extension Services administration, leadership, or organizational structure are barriers or facilitators to move healthy eating and active living PSE change work forward? c. What aspects of target rural communities or target settings where healthy eating and active living PSE changes are intended for are barriers or facilitators to moving this type of work forward? d. What challenges or opportunities do you as delivery agents encounter in moving forward healthy eating and active living PSE changes?
What types of resources are needed to effectively move healthy eating and active living PSE changes forward in rural communities? a. What are all the things that would be required to make it possible to implement healthy eating and active living PSE changes in all rural communities in the state of Louisiana?

The focus group discussion purpose was described to participants as useful for understanding what helps or challenges the complex rural PSE change work that LFCS practitioners are engaged in so that appropriate solutions could be identified. All LFCS practitioners present at the five regional trainings demonstrated interest in study participation and provided written informed consent (*n* = 40). One experienced qualitative researcher external to LFCS (BH) facilitated focus group discussions and a graduate research assistant (also external to LFCS; NP) took field notes (both female researchers). These sessions were audio recorded and conducted during a catered lunch hour, which served as study compensation.

Self-reported social and demographic characteristics of LFCS participants were not collected to reduce burden and to allow the full amount of time for the focus group discussions, which included between 6 to 9 practitioners across the five regions (most participants were female per field notes). After the initial focus group discussion, there was a request for an additional, anonymous mode to share information with researchers, so an online, optional Qualtrics survey following the semi-structured questionnaire (Table 1) was created and shared with participants after each regional focus group discussion. Four LFCS practitioners chose to share information with researchers in this way.

### Data analysis

Audio recordings of the focus group discussions were transcribed verbatim using an online service (Rev.com). To ensure data quality, a graduate research assistant (NP) compared focus group audio files to the transcriptions and adjusted for accuracy and removed any identifying information. The information shared via the optional Qualtrics survey was merged with corresponding regional transcript files, which were uploaded to a qualitative analysis software (Dedoose, version 9.0.85). Each transcript was deductively coded by two researchers independently (NP and BH or LB) following the 2022 CFIR [47] and using the constant comparison method (e.g., discussions and qualitative coding occur iteratively) [57]. Barrier and facilitator codes were also applied [47]. After initial coding, researchers met to discuss and reconcile discrepancies. Then, two researchers (NP and BH) reviewed all codes to ensure consistent application across all data and met to discuss any remaining discrepancies.

Qualitative saturation was assessed based on code application across the five regional transcripts [56]. Although all main 2022 CFIR domains and most of the constructs were applied among all five focus group discussions, indicating that the prominent barriers and facilitators reported below met saturation, there were a small number of 2022 CFIR constructs applied only among some regional transcripts. This may indicate that while there are mostly shared barriers and facilitators to delivering PSE changes across the state, certain regional nuances may require unique implementation consideration. For example, the more rural practice regions (Northwest and Northeast Louisiana) had a higher number of reported barriers compared to regions closer to larger metropolitan areas. To improve our understanding of results synthesis, a graduate research assistant traveled to all five LFCS regional trainings in 2022 as a form of member checking and presented on main study findings. There was noted agreement among LFCS practitioners regarding the study findings, despite these nuances.

Last, LFCS practitioners’ perspectives were often coded with multiple 2022 CFIR domains and constructs, representing the inter-connected nature of barriers and facilitators to implementing PSE changes in rural communities. Only ideas described more than once regarding the applied 2022 CFIR codes are reported below, as the constructs are considered categories (i.e., sets of similar content) requiring at least two items [58]. The number of codes represent the number of times each 2022 CFIR construct was mentioned as a barrier or facilitator. This approach was chosen rather than reporting the number of participants mentioning each construct, as focus groups (versus interviews) rely on group dynamics and an engaged discussion, and the number of mentions reveal the importance of the topic within the group’s discussion [59]. Further, quotes were used to highlight the similarities and differences between categories, enrich the analysis, and enhance transferability [58, 60].

## Results

LFCS practitioners across the five practice regions (*n* = 40) described more barriers (210 code applications) than facilitators (100 code applications) regarding implementing rural PSE changes. These in majority focused on 2022 CFIR *Inner* and *Outer Setting* domains, followed by *Individuals* and *Innovation*. Results are described below in the order of prominence (Table 2) and with supporting quotes to illustrate LFCS practitioners’ ideas and perceptions.

**Table 2:** Practitioners’ barriers and facilitators to healthy eating and active living policy, systems, and environmental (PSE) changes

2022 Consolidated Framework for Implementation Research (2022 CFIR) Domains and Constructs	2022 CFIR Domain and Construct Definitions	Barriers: Number of Codes^1^	Facilitators: Number of Codes^1^
**Inner Setting: Louisiana Cooperative Extension Services Family and Consumer Sciences (LFCS) (** ***n*** ** = 135)**			
Communications	Formal and informal information sharing practices supporting PSE implementation	28	21
Access to Knowledge & Information	Access to guidance or training on PSE implementation	26	10
Culture: Deliverer-Centeredness	Values, beliefs, and norms around caring, supporting, and addressing needs and welfare of LFCS practitioners	19	4
Culture: Learning- Centeredness	Values, beliefs, and norms around psychological safety and continual improvement to inform implementation	2	-
Available Resources: Funding	Availability of funding to implement PSE changes	16	5
Available Resources: Materials & Equipment	Availability of supplies to implement PSE changes	9	15
Structural Characteristics: Work Infrastructure	Organization of tasks and responsibilities supporting implementation of PSE changes	8	-
Structural Characteristics: Information Technology	Technological systems for communication, documentation, and data reporting, supporting PSE implementation	2	-
Incentive Systems	Tangible or intangible incentives, disincentives, or rewards supporting PSE implementation	7	-
**Outer Setting: Perceptions about the rural community, system, or state context (** ***n*** ** = 122)**			
Partnership & Connections	Connections with community partners or other organizations external to LFCS	44	24
Local Conditions	Economic, environmental, political, or technological conditions enabling PSE implementation	22	6
Financing	Availability of funding from external entities (e.g., grants, reimbursement) to implement PSE changes	15	4
Local Attitudes	Sociocultural values and beliefs enabling PSE implementation	11	9
Critical Incidents	Large-scale or unanticipated events that disrupt the outer setting and influence PSE implementation	4	4
Policies & Laws	Legislation, regulations, or professional group guidelines enabling PSE implementation	3	-
**Individuals: Characteristics of LFCS practitioners or other implementation team members (** ***n*** ** = 110)**			
Opportunity	Availability, scope, and power to fulfill role	35	-
Capability	Interpersonal competence, knowledge, and skills to fulfill role	26	-
Motivation	Commitment to fulfilling role	19	27
Need	Survival, well-being, or personal fulfillment needs to be met through PSE implementation	6	2
**Innovation: Healthy eating and active living PSE changes (** ***n*** ** = 67)**			
Innovation Complexity	PSE complexity (scope, number of connections or steps)	59	4
Innovation Adaptability	Potential to modify, tailor, or refine PSE changes	-	3

^1^Often LFCS practitioners’ ideas were coded as more than one 2022 CFIR construct. Codes are only reported if more than one application was captured

### CFIR inner setting

#### Communications

Organizational communication barriers were commonly reported (Table 2), although the nature of these barriers varied. In majority, LFCS practitioners described inconsistent communication streams as barriers based on the programs that funded their positions. For example, SNAP-Ed and the CDC High Obesity Program were described to have more robust communication structures for PSE changes than other programs:

“*As a non-SNAP-Ed employee, I don't get the Healthy Community’s emails that would be really supportive, nor am I on … the SNAP-Ed calls…* [that] *have success stories and you talk about what other people do*.” Communication barriers were also described regarding community PSE change implementation. Many LFCS practitioners believed that media communications about PSE changes should originate from rural community partners, instead of Extension, to build trust and support: “*So the other communities may say, I don't want to be a part of this because our church did this and we don't get the credit*.”

The timing of internal communications to support rural PSE changes were also, at times, a barrier:

“*That's why I just don’t ask questions because you get the response, ‘I said that six months ago’*.” Sometimes communication priorities were described as less important among LFCS practitioners amid other tasks:

“*I don't always have the time to send something to* [name] *so that we can get a press release out*.”

Other than having position support from a program prioritizing PSE changes (e.g., SNAP-Ed, CDC), which was a key communications barrier described above and the key communications facilitator, LFCS practitioners also considered the ability to connect with other LFCS practitioners as a facilitator. For example, this included the ability to connect with LFCS peers or Extension leadership that were experienced in implementing PSE changes: “*If I've had any questions, she's a phone call away. She's been very helpful*.”

#### Access to knowledge & information

LFCS practitioners noted a lack of knowledge/information at critical time points in implementing rural PSE changes, regarding knowing what resources are available, what rules or regulations pertain to a rural community setting, or who to ask for support (Table 2): “*And who do I ask? Where do I go from here?*” Further, policy change strategies were specifically a noted knowledge gap among some: “*I love examples of policy changes. Because we get it. We all know how to do a community garden now. We all know how to do a walk audit, stencil. We want policy examples. Because I don't know how I would do it*.” As stated above and throughout, access to knowledge/information was also a barrier that was often dependent on the programs supporting LFCS practitioners’ positions. This was also noted as the main facilitator (Table 2): “*So I work on the CDC health grant, and I feel so supported*.”

#### Culture

Organizational culture was also described as a barrier (Table 2). Some LFCS practitioners shared not feeling well supported by leadership, including work autonomy: “*At the state office, they're allowed to work a day at home of their flex time and I can't do that, but I'm out there doing the hard stuff, I think that has a lot to do with our morale*.” The idea that the position requirements for rural PSE changes (e.g., travel, forging and maintaining strong community ties) were not well aligned with leadership’s expectations or performance evaluations were also shared as barriers. Inconsistent support for implementing PSE changes, despite it being a required EBI among all LFCS practitioners, was a source of discontent: “*We're expected to, but I don't feel like we all get the full picture of what it actually is and what's expected of us*.”

Some LFCS practitioners described support at multiple levels (e.g., Regional Coordinators), as well as leadership’s understanding of the flexibility required for implementing rural PSE changes, as key facilitators: “*Sometimes our coalition wants to change the order of the steps and we have to be flexible. And so, we have been, and the state office has been understanding of that*.”

#### Resources

Limited organizational resources to implement rural PSE changes was another reported barrier (Table 2). Similarly, resource disparities were explained by the programs that supported LFCS practitioners’ positions: “*Just from an EFNEP standpoint, SNAP has funding to do certain PSE stuff that we don't have*.” This was also described as possibly causing tensions within communities, as explained by an LFCS practitioner who provided a community perspective: “*Well, what the heck? That parish got by without having to spend any money and they got a better project, and we spent money*.” Finding sources of funding to supplement limited internal funds or to overcome a lack of funding in general was a challenge: “*A lot of the things that we're encouraged to do, we can't fund. So, you are charged with finding these other funding sources*.” Additionally, insufficient materials or equipment for implementing PSE changes was a reported barrier: “*There is a lot of wear and tear on our personal vehicles… My vehicle can only hold so much. I don't want to place wet paint cans or stencils into my vehicle*.”

Consistent with noted barriers, facilitators were most often described as position support by a program with sufficient access to education, materials, and financial support: “*I like examples, tools, guidance, and especially funding*.” Providing resources to all LFCS practitioners regardless of program and creating methods to easily identify the available resources were described as potential facilitators for PSE changes (Table 2).

#### Structural characteristics

To a lesser extent, structural organizational barriers were described (Table 2). LFCS practitioners described having multiple organizational roles or splitting time among several programs that made balancing priorities difficult. Further, those with more education-focused responsibilities were described as left out of organizational PSE change efforts, although they were considered critical for facilitating community relationships for rural PSE changes: “*Sometimes they're not in the trainings that we have around PSE… but I think the trainings would help them see big pictures so that they could do more… Because they're the ones directly with the people and the people that are harder for us to reach*.” No facilitators were described for this 2022 CFIR construct (Table 2).

#### Incentive systems

At times, LFCS practitioners described a disconnect between position responsibilities and salaries/wages, regarding the effort required to overcome barriers and implement rural PSE changes: “*We don't make much money and our educators make even less. Yet, we are seen as leaders in the community making these things happen*;” and, “*We're expected to spend a lot of time outside of 8:00 to 4:30, but we're not compensated for it*.” No facilitators were described for this 2022 CFIR construct (Table 2).

### CFIR outer setting

#### Partnership & connections

The main barrier described by LFCS practitioners was difficulty initiating and sustaining community partner engagement in the coalition model used to initiate rural PSE changes (Table 2): “*It can be a challenge in those rural settings to find somebody and then find somebody who is committed for the long run to continue*.” This was also described to vary depending on the community and available resources: “*If they have a rec*[reation] *department, you're good to go. If they don't, good luck, you're starting from scratch and it's all on you and your coalition*.” Trust building in rural communities was also described as key for implementing PSE changes; however, mismatched resources among Louisiana parishes potentially diminish this trust: “*One particular partner said, we want the same things in the north that the south has… it appears unfair. It really does.*” Last, some described power imbalances regarding who is represented in coalitions (i.e., often less likely to include end-users of rural PSE changes) and ownership of PSE changes (i.e., LFCS rather than communities).

Identifying key partnerships in rural communities to improve the success of PSE changes was reported as a facilitator (Table 2). Remaining flexible and taking the time to build trust were considered critical: “*I think once you get their support, then you can do other stuff*”. Affiliation with the wider Extension organization, which also implements 4-H, a youth development program, was also important: “*Having the respect of the 4-H Office behind me has had its perks*”. LFCS practitioners being current or former residents of the rural parish and the tight-knit nature of rural communities/settings were at times also described as helpful for PSE changes: “*A lot of churches have a little bus, and they'll go pick people up. A lot of churches, they can ask for a love donation, love offering, or they have extra resources to be able to help ease the cost burden for people like me that, I'm not grant funded.*”

#### Local conditions

Often LFCS practitioners described limited resources and longstanding inequities in rural community settings as a barrier to implementing PSE changes (Table 2), for example: “*Also, I would say that there are a lot of barriers as far as in rural parishes, such as they may not have a grocery store like* [name] *said, they may not have a particular area that they're able to play in or walk in. So, you have to figure out certain locations that those projects can be implemented in*.” Also described was a conflict between not being able to “be political” in their professional positions and PSE changes that are inherently in conflict with local politics or perceptions: “*I don't know that we can* [enact policy changes] *because it becomes so political*”. To a lesser extent, hurricane occurrences were described as changing the population of rural communities due to displacement.

Rural community conditions were at times described as facilitators (Table 2). LFCS practitioners perceived high demand for PSE changes in rural communities given limited community resources and an opportunity to make a difference: “*Raising a family in a rural community, and then having this as my job as well, it really has opened my eyes, because there is huge impact in small places*.” Smaller population sizes were considered helpful for generating buy-in and establishing critical relationships for PSE changes: “*It's easier for me to do PSE work in the rural areas for me personally, it's a more tight-knit group, you can get in with elected officials and churches and people easier and it's just a lot more buy-in from them*.”

#### Financing

External funding to support PSE changes was described as a barrier (Table 2). Funding rules regarding what types of supports can be financed (“*EFNEP cannot pay or cover the expense of the paint and such, whereas SNAP can*”) as well as grant timelines or policies (e.g., overhead costs) compared to community goals or needs were reported challenges. This was considered pertinent given the expectation of LFCS practitioners to identify funding sources to close financing gaps for rural PSE changes. Facilitators were described as rural communities or partners that had the resources to sponsor or fund PSE changes (Table 2).

#### Local attitudes

Rural community members’ perceptions were at times a barrier to PSE changes (Table 2). LFCS practitioners described limited community awareness of LFCS programs at times (e.g., compared to other program areas such as 4-H) or a mismatch between the EBI and community members’ perceptions about acceptable ways to encourage rural healthy eating and active living: “*Greatest barrier is working with some smaller towns. Many are still ‘old school’ and aren't as warm to some PSE ideas*.” Local perceptions were sometimes also described as a facilitator (Table 2). As described among other constructs, the perceived ease of building local relationships due to the “tight-knit” nature of rural populations and the popularity of the 4-H program in rural areas helped to facilitate PSE changes.

#### Critical incidents

Although less prominent compared to other barriers, events such as hurricanes or the COVID-19 pandemic were reported (Table 2). Devastating impacts of major hurricanes were mentioned as disrupting opportunities for rural PSE changes, “*When the hurricane hit, a lot of things just… we're just trying to build back up*.” Additionally, the transition to virtual communication among partners during the early months of the COVID-19 pandemic was a barrier for rural areas that lacked internet access. However, the COVID-19 pandemic was at times described as a facilitator for rural PSE changes, given community leaders had more time to prioritize PSE changes during this period and some community members preferred the convenience of virtual meetings during and after the initial pandemic closures. This period also prompted a transition of practitioners in education-focused roles to initiate less complex PSE changes for the first time (i.e., stencils) (Table 2).

#### Policies & laws

There were a few regulatory barriers described and no facilitators (Table 2). Local regulations at times prevented the types of PSE changes allowed: “*I brought up stencils… and they immediately said, they said there was a policy against it*.”

### CFIR individuals

#### Opportunity

A primary barrier described among LFCS practitioners were the enormous demands (time and resources) for implementing rural PSE changes compared to the limited time available during the workday (Table 2). Further role delineation was a potential solution: “*So it's just one of those things where if we could use the nutrition educator model and hire professionals to do the direct education and have the agents focus on Healthy Communities, we could get a lot more done and we would have a better work life balance. But right now, we're trying to do it all and eventually some of these plates are going to drop*.” Rural community partners for PSE changes were also described as having limited time and, as such, LFCS practitioners needed to plan for connecting after normal working hours: “*I can't just roll up in their place of business like, ‘Hey, let's talk Healthy Communities real quick.’ I mean, they have things that they have to be doing too, so then I'm expecting them on top of me to come meet at 5:30 or 5:00 or whenever*.” No facilitators were described for this 2022 CFIR construct (Table 2).

#### Capability

LFCS practitioners mainly described the difference in professional scope regarding priority PSE changes highlighted by rural communities and their background and expert areas (Table 2): “*That's not my scope of practice and it never was. I'm not a facilities or planning person, so I don't have those contexts*.” Sometimes LFCS practitioners also described low confidence or capacity to identify key rural community partners that have the interest and leverage to help initiate PSE changes: “*It's like I'm constantly trying to figure out, even though this is not my job, I'm trying to figure out who can do this?*”. LFCS practitioners also wanted more training about larger-scale policy changes for rural healthy eating and active living. More general PSE trainings were also a noted need among some, especially those funded by programs not currently well supported for PSE changes (Table 2).

Further, sometimes LFCS practitioners described historical and structural issues in rural community settings as contributing to lower trust or capacity among community members to engage in PSE changes: “*Another thing that I have also noticed in rural communities, mine specifically, some of the community members they’re just discouraged about things because they normally feel like when people come into their towns with grants that is more about the numbers. Like, ‘Oh, well people always come here. They always promise the things. They always say, we’re going to do things. They get the numbers and then they leave*’”. No facilitators were described for this 2022 CFIR construct (Table 2).

#### Motivation

Fewer barriers than facilitators were described for this 2022 CFIR construct (Table 2). Noted barriers primarily centered on LFCS practitioners’ efforts to engage community members in implementing PSE changes initially and over time: “*Well, and then it becomes disappointing when you worked really hard on a project and you can’t stay with that project forever, you’ve got to at some point turn it over to them. And then when you step out, it just stops*.” To a lesser extent, low motivation for LFCS practitioners to travel to the more rural areas of the state to implement PSE changes was also described: “*But they do work the area, it’s just on paper*.” The primary facilitator was described by LFCS practitioners as buy-in from a community leader or champion, which helped to initiate complex PSE changes, “*You have to have that one community champion that really wants this to happen*.” Also, personal attributes were also described as facilitators for PSE changes, such as being vocal regarding asking questions and making community connections. Importantly, LFCS practitioners’ local ties to rural communities was a facilitator, “*Having grown up with public officials and business owners has made it easier to implement the projects since they already know and respect me*.”

#### Need

There were a few instances where LFCS practitioners described not reaching the rural populations that could most benefit from structural changes to promote healthy eating and active living (Table 2): “*I think sometimes we’re not really reaching the people who actually would be using the environmental changes… which I think is who we really need to be reaching in order for sustainability*.” Also described was the reality that PSE changes may not be a priority in some rural communities amid other pressing challenges: “*They’re not worried about putting in a new playground right now. They have bigger fish to fry*.” Although facilitators were less common (Table 2), LFCS practitioners described rural community demand for PSE changes and the community coalition model as beneficial for identifying priority PSE changes, “*What we finally drilled down to was they’re in a food desert, lack of transportation, lack of community, lack of access to physical activity. We built our initiatives around those needs*.”

### CFIR innovation

#### Innovation complexity

LFCS practitioners described the challenge of implementing rural PSE changes given they span multiple sectors, rely on invested partnerships, and require specialized knowledge (Table 2). For example, this complexity was described as difficult to grasp and to communicate: “*I feel like happens and in my parishes and maybe in you all’s too, but people don't know what we doing. It took years I think for even the AgCenter to really understand*.” However, LFCS practitioners often described easier-to-implement PSE changes, such as stencils to encourage physical activity: “*Just in our every day and everything else that we do, we stick to the easier ones*.” Systems changes for healthy eating and active living were also considered easier, given the reliance on building relationships to facilitate these changes, which LFCS practitioners considered themselves as well positioned to initiate (Table 2).

#### Innovation adaptability

No barriers were described for this 2022 CFIR construct (Table 2). Some LFCS practitioners believed rural PSE changes to be adaptable to local community settings and needs: “Y*ou can decide, oh, I can use this, but I'm going to tweak it just a little bit to make it work for my people*.”

## Discussion

The purpose of this study was to identify barriers and facilitators to the implementation and eventual scaling of PSE changes in rural Louisiana parishes/communities from public health practitioners’ perspectives. Importantly, barriers were present at multiple levels of influence, including the LFCS organization (*Inner Setting*), rural Louisiana communities (*Outer Setting*), LFCS practitioners (*Individuals*), and PSE changes (*Innovation*). While the complexity of healthy eating and active living PSE changes are unlikely to be modified given the focus on dismantling structural barriers to health, results indicate there are opportunities to improve rural PSE change implementation within and between the LFCS Extension organization and community partners.

Mainly, efforts to overcome organizational communication barriers and inconsistent access to information dependent on program funding as described by LFCS practitioners may help to facilitate rural PSE changes. New funding streams and strategies to align organizational perspectives and expectations for PSE changes as a priority EBI among all levels of Extension and LFCS are also likely needed. Building capacity and support for rural PSE changes by introducing new training opportunities and providing professional incentives will also likely improve implementation outcomes. Using health equity frameworks as an added tool to guide rural PSE change process could also help build opportunities for LFCS practitioners to reach all rural community members. These efforts should build on noted assets, including LFCS practitioners’ professional practice and relationship-building expertise, their motivation to make a difference in rural Louisiana communities, and the built-in organizational and community supports already available for many LFCS practitioners to implement rural PSE changes.

The results presented here confirm contextual factors that likely influence the success or failure of PSE change implementation that have been identified through similar work. For example, inquiries in other states have also found that Extension practitioners are motivated to implement PSE changes, although have lower confidence and skills for PSE changes in comparison to implementing direct education [24–26]. As well, other studies found limited training and PSE complexity as key barriers [24, 25, 28]. Partnerships and connections (e.g., working with community coalitions) has been noted as either a facilitator or a barrier in other work [26, 27], although it has not been described as *both *a barrier and facilitator as it was here. This may indicate that multi-sector collaborations needed for PSE changes are a new practice for some Extension practitioners, and successes and challenges can vary. This may also indicate the need for more tailored capacity-building strategies for working with community coalitions, as was suggested in an Extension-based study conducted in Arkansas [26].

There were a few novel findings of this study. First, inconsistent communications for PSE changes have not been previously reported among similar research conducted in other states, which may be due to the study populations (e.g., those that focused only on nutrition educators/paraeducators funded through SNAP-Ed or EFNEP rather than differences within a state Extension system) [25, 39–43]. This highlights an opportunity to improve organizational collaboration and communication to leverage multiple funding streams. Second, LFCS practitioners’ scope and power to fulfill their role (the *Opportunity *construct) was a unique finding of this study. Specifically, the mismatch between complex rural PSE change implementation and a traditional 9-to-5 schedule was a barrier. While LCES practitioners’ degree of autonomy and status (e.g., staff vs. faculty) varies by state [61], flexible scheduling has been suggested for reaching diverse community members (e.g., versus reaching primarily youth or retirees during daytime hours) and decreasing practitioner burnout and turnover [62, 63]. These are key strategies to consider for overcoming prominent contextual barriers to rural PSE changes.

The results of this study provide insights into several future directions for improving the implementation of multi-level PSE changes within public health practice settings. An in-depth contextual inquiry to understand local implementation barriers and facilitators, as presented here, is key to selecting implementation strategies to improve the adoption, implementation, sustainment, and eventual scaling of PSE changes [32, 33, 36, 64–68]. Using these results as a guide, researchers are undergoing efforts to understand implementation strategy priorities for rural PSE changes in collaboration with LFCS practitioners. While Extension is a complex system [46] there are many commonalities in systems and structures across states. Therefore, results and potential solutions identified because of this study may be applicable beyond the LFCS practice setting, given the in-depth qualitative inquiry approach used following a standard implementation science framework [44, 45].

Robinson & Damschroder (2023) introduced a pragmatic context assessment tool based on barrier and facilitator findings following CFIR, to rapidly assess local factors in clinical settings that might influence EBI adoption, implementation, and sustainment [69]. A similar approach is warranted regarding PSE changes within U.S. Extension systems to conserve limited public health resources. For example, this study required substantial travel and professional time and resources over the course of a year. It is unlikely the same level of time and resources are required to explore barriers and facilitators to PSE changes in other settings (e.g., urban Louisiana communities, other state Extension systems). A pragmatic assessment method [69] that bases on the results of this in-depth contextual inquiry could be created to explore this assumption. This approach to contextual inquiry and implementation strategy development could also be used as a model for other public health systems in the U.S. or abroad.

### Strengths and limitations

There are several strengths and limitations of this work to note. This study represented a rigorous qualitative contextual inquiry among implementation science and Extension partners to identify multi-level barriers and facilitators to LFCS practitioners’ implementation of rural PSE changes. The focus on the statewide LFCS network, rather than among practitioners supported by certain funding streams (e.g., SNAP-Ed only), is also a strength. However, there are limitations. Barriers may have been underreported given the expressed discomfort among some participants regarding sharing information in a focus group discussion setting. Although researchers were external to LFCS and additionally sought to overcome this limitation by providing an anonymous survey response option for additional feedback, some nuance may have been lost. Further, qualitative data saturation was not fully achieved, given some unique barriers or facilitators were only shared among some practice regions. Project resources prevented additional travel to further explore these concepts; however, many LFCS practitioners did comment on the unique qualities of Louisiana regions multiple times during the study. This may indicate that barriers and facilitators (and associated implementation strategies) may need to be tailored to region. Moving forward, we recommend future pragmatic context assessment methods provide opportunities for open-ended responses to understand the potential for contextual variations by region or setting.

## Conclusions

This study examined Extension practitioners’ multi-level barriers and facilitators to implementing PSE changes in rural Louisiana communities. Future work includes tailoring relevant implementation strategies to overcome noted barriers, which could include improved organizational communication and information access, new funding streams and strategies, and enhanced capacity-building support. In particular, findings of this study suggest improved organizational communication and structures across all levels of parish, regional, and state faculty and administration could be explored as a way to both bridge the communication disconnect and address cultural barriers that impact morale. Finally, rapid contextual inquiry processes could be used to determine which barriers and facilitators (and associated implementation strategies) are relevant in other state Extension systems. Enhancing the adoption, implementation, and sustainability of healthy eating and active living PSE changes can remove structural barriers and improve community members’ health practices.

## Data Availability

Not applicable.

## Abbreviations

PSE: Policy, Systems, and Environmental
LFCS: Louisiana Cooperative Extension Service Family and Consumer Science
CFIR: Consolidated Framework for Implementation Research
U.S.: United States
SNAP-Ed: Supplemental Nutrition Assistance Program Education
EFNEP: Expanded Food and Nutrition Education Program
CDC: Centers for Disease Control and Prevention
EBI: Evidence-Based Intervention
FCS: Family and Consumer Science
LSU: Louisiana State University
SUAREC: Southern University Agricultural Research and Extension Center
